# Mean Bone Material Strength Index Values for Women are Lower Than Those for Men: Data from a Single Geographical Location

**DOI:** 10.1007/s00223-023-01133-9

**Published:** 2023-09-04

**Authors:** Kara L. Holloway-Kew, Pamela G. Rufus-Membere, Kara B. Anderson, Jacob W. Harland, Adolfo Diez-Perez, Mark A. Kotowicz, Julie A. Pasco

**Affiliations:** 1 IMPACT, The Institute for Mental and Physical Health and Clinical Translation, School of Medicine, Deakin University, Geelong, Australia; 2 Department of Internal Medicine, Hospital del Mar-IMIM, Autonomous University of Barcelona and CIBERFES, Instituto Carlos III, Barcelona, Spain; 3 Barwon Health, Geelong, Australia; 4 Department of Medicine, The University of Melbourne, Western Health, St Albans, Australia; 5 Department of Epidemiology and Preventive Medicine, Monash University, Melbourne, Australia

**Keywords:** Impact microindentation, Bone material strength index, Men, Women

## Abstract

Bone material strength index (BMSi) values are obtained using impact microindentation, which assesses the ability of bone to resist indentation. Differences in BMSi between men and women are unclear, and to date, BMSi sex differences have not been compared for individuals from the same population. Therefore, we compared BMSi values for men and women drawn from the same geographical location in Australia. Participants (n = 220) were from the Geelong Osteoporosis Study. BMSi was measured, following international published guidelines, using an OsteoProbe for participants at recent follow-up phases (women 2022–2023 and men 2016–2022). Women (n = 55) were age matched to men (n = 165) in a 1:3 ratio. A two-sample t test was used to determine the intergroup difference in mean BMSi. Linear regression was also performed, adjusting for weight and height. Median (IQR) ages for men and women were 67.0 (61.7–71.5) and 67.4 (62.0–71.2) years (p = 0.998). Men were heavier (81.0 ± 10.9 vs 71.0 ± 13.9 kg, p < 0.001) and taller (173.9 ± 6.4 vs 161.5 ± 7.5 cm, p < 0.001) than women. Mean (± SD) BMSi for women (75.7 ± 7.4) was lower than for men (82.8 ± 6.8) (p < 0.001). The difference persisted after adjustment for weight and height (mean ± SE: 76.5 ± 1.1 vs 82.5 ± 0.6, p < 0.001). Given the higher fracture risk observed for women, the higher mean BMSi values in men are consistent with cross sectional data suggesting this measure may be useful in fracture prediction.

## Introduction

Impact microindentation (IMI) is a relatively new technique that uses a handheld device known as the OsteoProbe [1] to assess fracture resistance of cortical bone at the mid-tibia. The device measures the indentation distance of the bone and compares it to the indentation distance of a polymethyl methacrylate reference material [2]. The ratio of these two indendation distances is then expressed as a unitless value called Bone Material Strength Index (BMSi).

Although sex differences in bone mineral density and fracture risk have been clearly reported [3–5], differences in BMSi between men and women are unclear. However, the recently published international healthy reference intervals suggest that there may be a difference between men and women [6]. To date, BMSi sex differences have not been compared for individuals from the same population, which is important, as differences have been reported between women from different geographical regions, namely Norway and Spain [7]. Therefore, in this study, we compared BMSi for men and women matched for age and drawn from the same geographical location in Australia.

## Methods

### Participants

Participants were from the Geelong Osteoporosis Study [8], a longitudinal cohort study situated in south-eastern Australia. Data for this study were derived from the most recent follow-up phases, which were 2022–2023 for women and 2016–2022 for men. This study included 55 women, who were age-matched to men (n = 165) in a 1:3 ratio, resulting in a total of 220 participants.

### Impact Microindentation (IMI)

IMI measurements to determine BMSi were performed following international recommended guidelines [9] using an OsteoProbe device (Active Life Technologies, Santa Barbara, CA, USA). The same device was used for both men and women. Measurements were performed on the mid-tibia, at the midpoint from the medial border of the tibial plateau to the distal edge of the medial malleolus. Briefly, local anaesthetic was applied to the measurement area, and then the probe tip was inserted through the skin. The operator then performed the measurements by pressing down the outer housing of the device. As reported previously [10], participants experienced minimal discomfort during measurements.

The first measurement for each participant was systematically discarded. This is because the first measurement is often affected by insufficient penetration through the periosteum. Following this, at least 8 measurements were performed, and between each one, the device tip was moved approximately 2 mm. Measurements were considered invalid if they appeared outside the “green zone” area indicated by the software. Additionally, measurements were removed if the operator reported abnormal bone “texture” while performing the measurements.

During the relevant follow-up phases of the Geelong Osteoporosis Study, there were four trained operators performing IMI measurements. However, almost three quarters of the measurements (73.6%) were performed by one operator (PR-M). The coefficient of variation (CV) for microindentation was 2% for repeated measures. Precision was calculated as the mean (expressed as %) of SD/mean for two sets of indentations for 10 participants.

### Other Data

Weight and height were measured to the nearest 0.1 kg and 0.1 cm, using electronic scales and a Harpenden stadiometer, respectively. Body mass index (BMI) was calculated as weight(kg)/height(m)^2^. Participants self-reported their use of anti-fracture medication (e.g. bisphosphonates, other antiresorptives).

### Statistical Analyses

Participant characteristics were described using means and standard deviations (SD) or medians with interquartile range (IQR) as appropriate. A two-sample t test was used to determine unadjusted differences in mean BMSi between men and women. Additionally, linear regression analyses adjusting for weight and height were performed. A sensitivity analysis was also performed excluding those taking anti-fracture medications. Analyses were completed using Minitab (Minitab, version 19, State College, PA, USA) and Stata (Version 17. StataCorp. 2017. Stata Statistical Software: Release 17. College Station, TX: StataCorp LLC).

## Results

### Descriptive Characteristics

Table 1 shows the participant characteristics. Men were heavier and taller than women. Despite this, BMI was similar for men and women.

**Table 1:** Participant characteristics

	Men (n = 165)	Women (n = 55)	p value
Age (years)	67.0 (61.7–71.5)	67.4 (62.0–71.2)	0.998
Weight (kg)	81.0 ± 10.9	71.0 ± 13.9	< 0.001
Height (cm)	173.9 ± 6.4	161.5 ± 7.5	< 0.001
Body mass index (kg/m^2^)	26.8 ± 3.1	27.2 ± 4.4	0.551
Bone material strength index	82.8 ± 6.8	75.7 ± 7.4	< 0.001
Anti-fracture medication use	5 (3.0)	3 (5.5)	–

Data presented as mean ± SD, median (IQR) or n(%) as appropriate

### Differences in Bone Material Strength Index

**Fig. 1:**
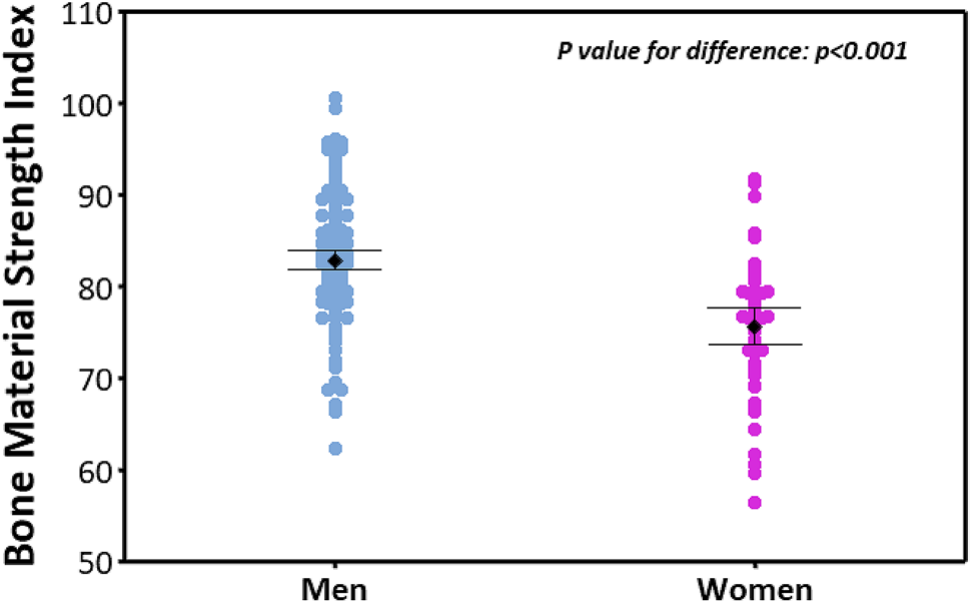
Bone material strength index values for men and women

The mean (± SD) BMSi for women was lower than for men (Table 1, Fig. 1, p < 0.001). After adjustment for weight and height, this association persisted. For women, adjusted mean BMSi was lower than that for men (mean ± SE: 76.5 ± 1.1 vs 82.5 ± 0.6, p < 0.001).

In the sensitivity analysis, there were eight participants (5 women, 3 men) taking an anti-fracture medication. Exclusion of these participants did not change the results; women had lower BMSi in unadjusted (mean ± SD: 75.4 ± 7.3 vs 82.8 ± 6.9, p < 0.001) and adjusted (mean ± SE: 76.1 ± 1.2 vs 82.6 ± 0.6, p < 0.001) analyses.

## Discussion

This study indicated that mean BMSi was higher for men compared to women drawn from the same population, independent of differences in weight and height.

These results are in agreement with a recently published study describing reference intervals for men and women from multiple international sites [6]. The study reported BMSi values of 84.4 ± 6.9 for men and 79.0 ± 9.1 for women (p < 0.001). These values are similar to that reported in our study, however, our study included age-matched individuals drawn from the same underlying population. This is important, as some differences have been reported between different geographical areas [7].

Many other previous studies including IMI measurements have included only one sex, usually women. However, a few have included both men and women. One study measured 50 individuals with human immunodeficiency virus (HIV) and 35 healthy volunteers [11]. Among individuals with HIV, men had a higher BMSi than women (median (IQR): 85 (83–87) vs 80 (77–83); p < 0.001). However, among the control group, no differences between men and women were observed (median (IQR): 92 (88–96) vs 89 (86–93), p = 0.07). The reason for differences between our study and this previous study could include that the control group were volunteers, whereas the participants in our study were unselected and derived from the general population. Additionally, the median age for individuals in our study was higher (approximately 67 years vs 36 years) and thus our study may have captured greater bone loss and microarchitectural deterioration that occurs at menopause in women. In another study that included 37 individuals with primary hyperparathyroidism [12], there was no difference in mean BMSi between men and women (79.6 ± 4.4 vs 77.7 ± 6.1, p = 0.404). However, when compared to controls (n = 37), those with primary hyperparathyroidism had lower mean BMSi (78.2 ± 5.7 vs 82.8 ± 4.5, p < 0.001). Again this is different to what was reported in our study, however, the sample size for the study was small (9 men, 28 women) and consequently there may not have been sufficient power to observe any differences. Additionally, no differences were observed between men and women with primary hyperparathyroidism, but the presence of the disease may have resulted in lower BMSi, regardless of sex.

There are also several studies that have included men and women who had sustained a fracture. Two of these studies included participants with fragility fracture compared to individuals without fracture, and both studies reported no differences between men and women [13, 14]. The third study compared participants with high and low trauma fractures, also reporting no differences between men and women [15]. Similar to the study including individuals with primary hyperparathyroidism, it is possible that fracture status has a larger impact on BMSi value than sex. However, the sample sizes in some of these studies were small and thus, larger studies would be needed to confirm this possibility.

Only one previous study has examined associations between risk of incident fracture and BMSi [16]. The study included 647 women aged 75 to 80 years, and reported that, unexpectedly, higher BMSi was associated with an increased risk of fracture. Further studies are needed to replicate this observation, as well as investigate whether there are sex differences in the association between BMSi and incident fracture.

Our study has some strengths and limitations. One major strength is that the men and women were from the same population, age-matched, and not selected on the basis of disease. Additionally the OsteoProbe operators and device were the same for both groups. The sample size was sufficient, and a power analysis showed the study had > 90% power to observe differences in BMSi between men and women. One limitation is that we are unable to provide any longitudinal data, to determine if the differences observed in BMSi between men and women also translate to a difference in fracture risk.

## Conclusion

These data support previous work that showed differences in BMSi between men and women, using pooled data from different geographical settings. This also contributes to baseline data for investigating the ability of BMSi to predict sex-specific fracture risk. Given the higher fracture risk observed among women, the higher mean BMSi values in men is consistent with cross sectional data and may explain, in part, why fracture risk is lower in men than women.
